# Electronic Structures and Transition Properties of BeSe and BeTe Molecules

**DOI:** 10.1021/acsomega.1c03170

**Published:** 2021-11-07

**Authors:** Israa Zeid, Nayla El-Kork, Mohamed Farjallah, Hela Ladjimi, Hamid Berriche, Mahmoud Korek

**Affiliations:** † Faculty of Science, Beirut Arab University, P.O. Box 11-5020, Beirut 1107 2809, Lebanon; ^‡^ Physics Department, Khalifa University, P.O. Box 127788, Abu Dhabi 51133, UAE; § Laboratory of Interfaces and Advanced Materials, Faculty of Science, University of Monastir, Monastir 5019, Tunisia; ∥ Department of Mathematics and Natural Sciences, School of Arts and Sciences, American University of Ras Al Khaimah, P.O. Box 10021, Ras Al Khaimah 10021, UAE

## Abstract

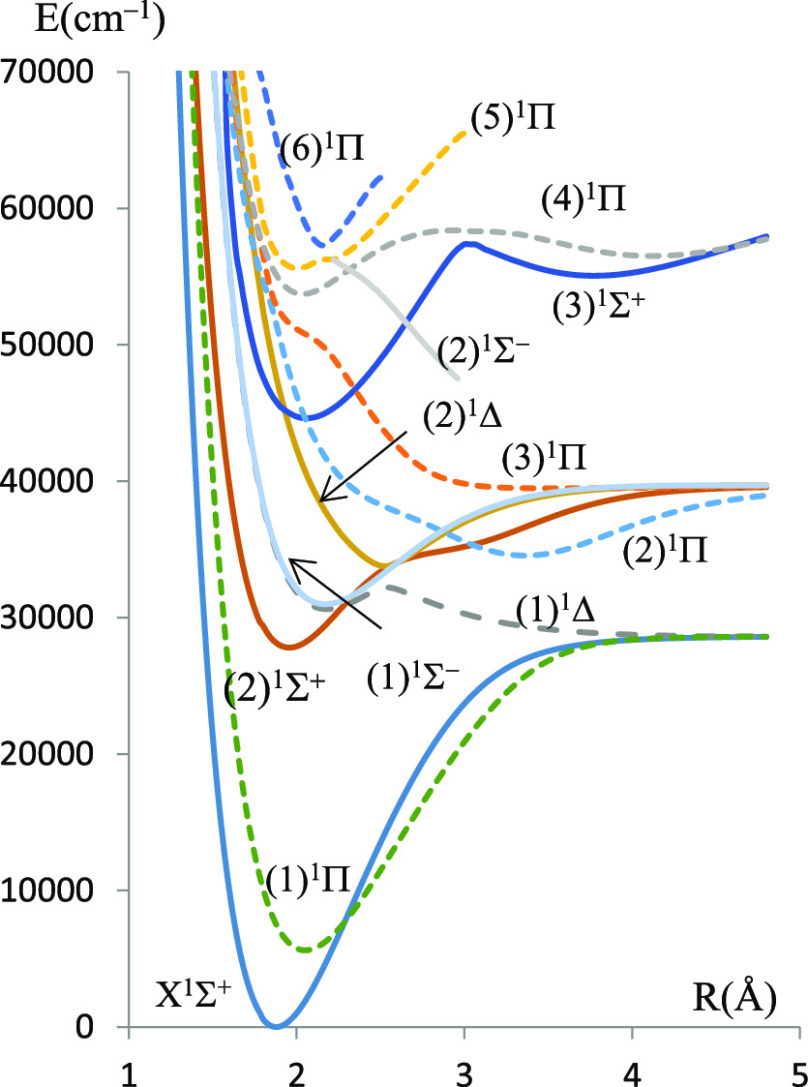

The electronic structure of BeSe and BeTe molecules has been investigated using the *ab initio* CASSCF/(MRCI + Q) method at the spin-free and spin-orbit level. The potential energy curves, the permanent dipole moment, the spectroscopic constants *T*_e_, *R*_e_, ω_e_, and *B*_e_, and the dissociation energy *D*_e_ are determined in addition to the vertical transition energy T_v_. The molecules’ percentages of ionic character are deduced, and the trends of the spectroscopic constants of the two molecules are compared and justified. A ro-vibrational study is performed using the canonical function approach to calculate the constants *E*_v_, *B*_v_, and *D*_v_ and the turning points *R*_min_ and *R*_max_. All the ground-state vibrational levels have also been investigated. The radiative lifetimes of vibrational transitions among the electronic ground states are also discussed. The results for BeSe have been compared with the previously published data while those for BeTe molecules are presented here for the first time.

## 1 Introduction

Solid-state beryllium chalcogenides (BeS, BeSe, and BeTe) exhibit several interesting physical properties related to their electronic structure; they present high bonding energy, hardness, and unusual electronic, mechanical, thermal, and optical properties. They are very interesting candidates for optoelectronic devices in the blue and UV spectral regions because of their high band gap energies.^1−10^

The molecule BeS has been studied experimentally and theoretically in the gaseous state.^11,12^ Its electronic properties have been found to be very helpful in the advancement of attosecond physics and the exploration of the dynamical motion of electrons.^13,14^ Also, the BeSe molecule has been the subject of the study conducted by Larbi et al. in 2021 where it is investigated via MRCI + Q in singlet, triplet, and quintet multiplicities.^15^ The molecule BeTe, on the other hand, has not been studied either experimentally or theoretically yet. Because of this missing data and as a continuation to our work on Be compounds,^16^ we present in this work a theoretical study on BeSe and BeTe compounds. We use CASSCF/MRCI *ab initio* calculations for these two molecules to calculate their potential energy curves, spectroscopic constants, dipole moments, main ro-vibrational parameters, and transition rates/radiative lifetimes.

## 2 Computational Approach

The singlet, triplet, and quintet electronic states of the molecules BeSe and BeTe, in the representation ^2s+1^Λ^±^, have been studied in the present work. We used the state-averaged complete active space self-consistent field (CASSCF) via the multireference configuration interaction (MRDSCI) with Davidson correction method. The high-level accuracy computational chemistry program MOLPRO^17^ has been used, taking advantage of the graphical user interface GABEDIT.^18^

The beryllium atom Be (of four electrons) was treated in all electron schemes using the correlated consistent polarized valence five zeta cc-pV5Z basis set for s, p, and d functions^19^ while the Se atom was treated as a system of 6 valence electrons and 28 electrons within the core using the effective core potential ECP28MWB basis set^20^ for s and p functions. Similarly, the Te atom was treated as a system of 6 valence electrons and 46 electrons within the core using the effective core potential ECP46MWB basis set^20^ for s and p functions. The active space in the *C*_2v_ symmetry group then contains 5σ (Be: 2s, 2p_0_, 3s, and 3p_0_ and Se: 4p_0_), 2π (Be:2p ± 1 and Se: 4p ± 1), and 0δ (Be:0 and Se:0) and 7σ (Be: 2s, 2p_0_, 3s,3p_0_, 3d_0_, and 3d + 2 and Te: 5p0), 4π (Be:2p ± 1,3p ± 1, and 3d + 1 and Te: 5p ± 1), and 1δ (Be:3d – 2 and Te:0) orbitals distributed into the irreducible representation as 5a1, 2b1, 2b2, and 0a2 and 7a1, 4b1, 4b2, and 1a2, which correspond to [5,2,2,0] and [7,4,1,1] for the molecules BeSe and BeTe, respectively.

We checked for the quality of the basis sets that we used by comparing the values that we obtained for the lowest energy at each dissociative asymptote with those obtained experimentally from the atomic spectra database (NIST)^21^ (Table 1). The relative differences that we obtain range between 0.01% ≤ Δ*E*/*E* ≤ 13.2% for the two considered molecules. This overall good relative error ensures the accuracy of our calculated data. Also, the electronic structure of BeSe and BeTe has been investigated using the ECP28MDF and ECP46MDF basis set^22^ for Se and Te, respectively, in the spin-free and spin-orbit representation while the basis of Be is kept as cc-pV5Z but the *f* function is introduced.

**Table 1:** Lowest Dissociation Limits of BeSe and BeTe Molecules

dissociation limit of atomic levels Be + Se	dissociation energy limit of BeSe levels (cm^–1^)	molecular states of BeSe	total dissociation energy limit of Be + Se atoms (cm^–1^)	relative error (%)
Be (1s^2^2s^2^, ^1^S) + Se (4s^2^4p^4^, ^3^P)	0^a^	(1)^3^Π, (1)^3^Σ^–^	0^b^	0.0
Be(1s^2^2s^2^, ^1^S) + Se (4s^2^4p^4^, ^1^D)	11,041^a^	X ^1^Σ^+^, (1)^1^Π, (1)^1^Δ	9576^b^	13.2
Be (1s^2^2s2p, ^3^P^0^) + Se (4s^2^4p^4^, ^3^P)	21,980^a^	(2)^1^Σ^+^, (1)^1^Σ^–^, (2)^1^Π, (3)^1^Π, (2)^1^Δ, (2)^1^Σ^–^ (1)^3^Σ^+^, (2)^3^Π, (3)^3^Π,(2)^3^Σ^–^, (3)^3^Σ^–^,(1)^3^Δ (1)^5^Σ^+^, (1)^5^Π, (2)^5^Π,(1)^5^Σ^–^, (2)^5^Σ^–^,(1)^5^Δ	21,978^b^	0.01

dissociation of atomic levels Be + Te	dissociation energy limit of BeTe levels (cm^–1^)	molecular states of BeTe	total dissociation energy limit of Be + Te atoms (cm^–1^)	relative error (%)
Be (1s^2^2s^2^, ^1^S) + Te (5p^4^, ^3^P)	0^a^	(1)^3^Π,(1)^3^Σ^–^	0^b^	0.0
Be (1s^2^2s^2^, ^1^S) + Te (5p^4^, ^1^D)	9911^a^	X ^1^Σ^+^, (1)^1^Π,(1)^1^Δ	10,557^b^	6.1
Be (1s^2^2s2p, ^3^P^0^) + Te (5p^4^, ^3^P)	22,074^a^	(2)^1^Σ^+^, (1)^1^Σ^–^, (2)^1^Π, (3)^1^Π, (2)^1^Δ (1)^3^Σ^+^, (2)^3^Π, (3)^3^Π,(2)^3^Σ^–^, (3)^3^Σ^–^,(1)^3^Δ (1)^5^Σ^+^, (1)^5^Π, (2)^5^Π,(1)^5^Σ^–^, (2)^5^Σ^–^,(1)^5^Δ	21,978^b^	0.4

a Present work using the ECP28MWB and ECP46MWB basis set for Se and Te, respectively.

b Experimental values from the NIST atomic spectra data base.

## 3 Results and Discussion

### 3.1 Potential Energy Curves, Permanent Dipole Moment, and Ionic Character *f*_ionic_

**Figure 1:**
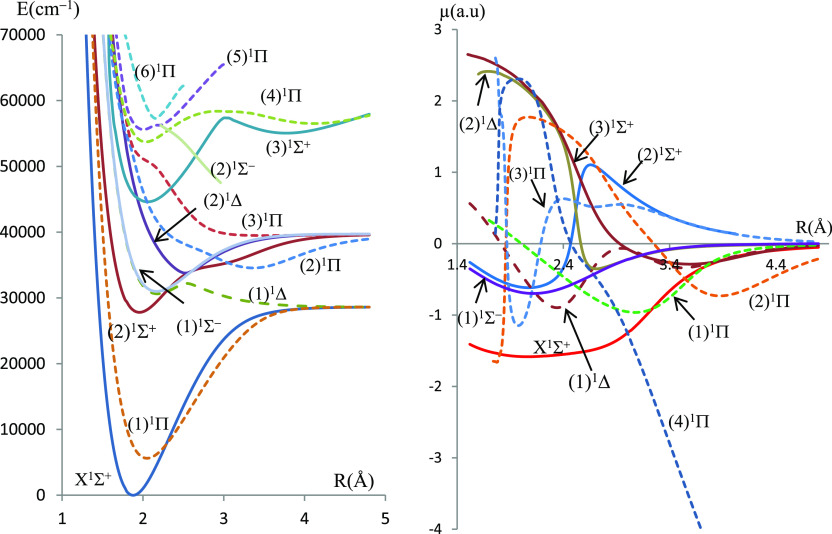
Potential energy curves and permanent dipole moment curves of the singlet states of BeSe molecules investigated using ECP28MWB for Se.

**Figure 2:**
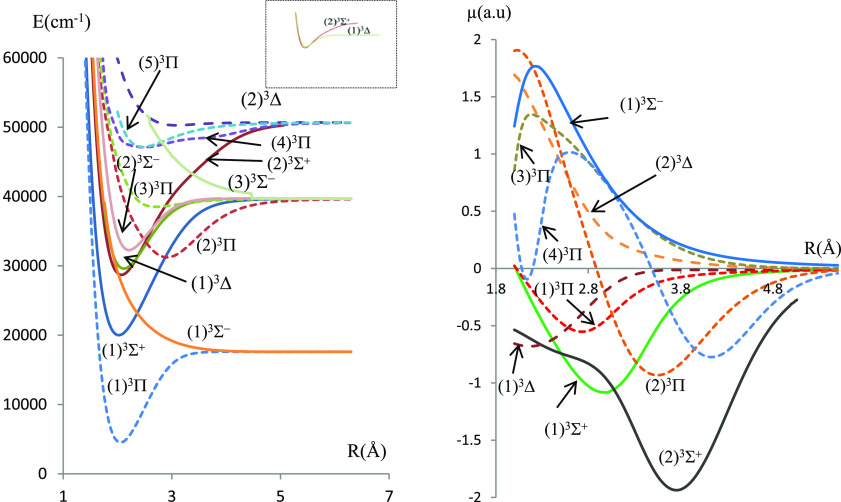
Potential energy curves and permanent dipole moment curves of the triplet states of BeSe molecules investigated using ECP28MWB for Se.

**Figure 3:**
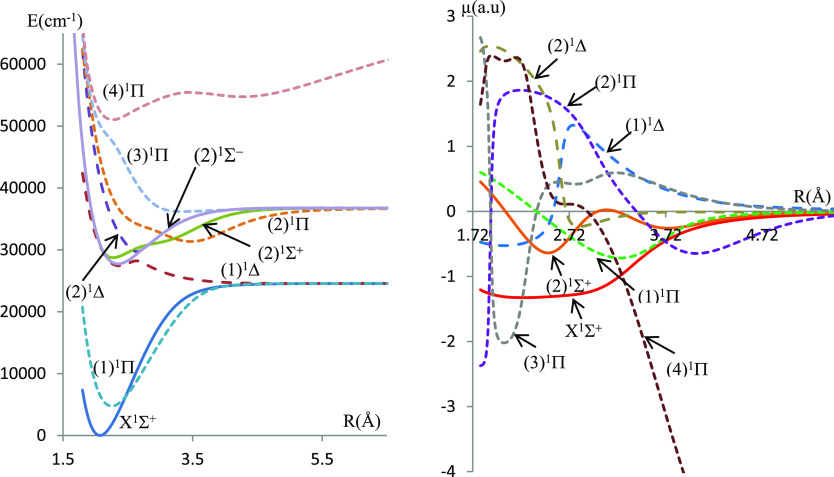
Potential energy curves and permanent dipole moment curves of the singlet states of BeTe molecules investigated using ECP46MWB for Te.

**Figure 4:**
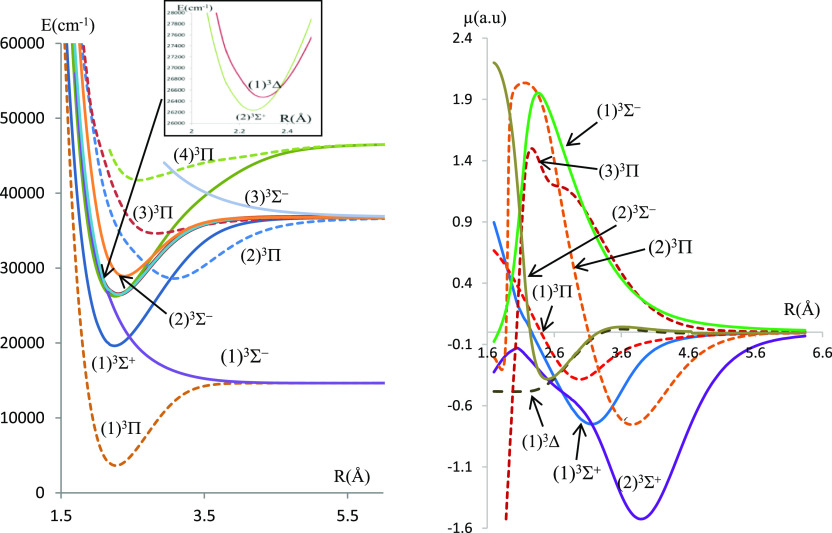
Potential energy curves and permanent dipole moment curves of the triplet states of BeTe molecules investigated using ECP46MWB for Te.

In the present work, electronic states in singlet, triplet, and quintet multiplicities have been investigated for the molecules BeSe and BeTe, respectively. The potential energy and the dipole moment curves for the singlet and triplet states calculated using the ECPMWB basis set for Se and Te are given in Figures 1–4 while those related to the quintet states are given in figures (FS1–FS4) in the Supporting Information.

The molecular dissociation asymptotes are given in Table 1. Some of the singlet electronic states result from the combination of a singlet Be atom with a singlet chalcogenide atom, and some of the triplet electronic states result from the combination of a singlet Be atom with a triplet chalcogenide atom. On the other hand, singlet, triplet, and quintet states that correlate in the same asymptotic limit of dissociation result from a triplet Be atom combined with a triplet chalcogenide atom.

The deep potential wells (large *D*_e_ values) for most electronic states are evidence of the dominant attractive forces over the repulsive ones, at equilibrium, in this type of molecules. The corresponding values of dissociation energies *D*_e_ are given in Table 2.

**Table 2:** Spectroscopic Constants of the Molecules BeSe and BeTe Calculated Using ECP28MWB and ECP46MWB for Se and Te, Respectively

BeTe							
state (^2S+1^Λ)	*T*_e_ (cm^–1^)	*T*_v_ (cm^–1^)	*R*_e_ (Å)	ω_e_ (cm^–1^)	*B*_e_ (cm^–1^)	*D*_e_ (cm^–1^)	μ_e_ (a.u)
X^1^Σ^+^	0	0	2.070	741.4	0.467	24,565	1.315
(1)^3^Π	3656	5697	2.266	532.3	0.389	11,014	0.154
(1)^1^Π	4746	6713	2.252	580.9	0.395	19,823	0.140
(1)^3^Σ^+^	19,627	21,337	2.248	558.5	0.394	17,116	0.027
(1)^1^Δ	27,420	30,820	2.366	452.5	0.358	2848	0.426
(2)^3^Π	28,617	42,146	3.083	315.8	0.210	8028	0.065
(3)^3^Π	34,639	45,844	1.495	464.4	0.896	2103	1.154
(4)^3^Π	41,739		2.591	321.0	0.298	4802	1.878
(4)^1^Π	51,037	52,585	2.267	433.1	0.390	13,461	2.165

BeSe							
state (^2S+1^Λ)	*T*_e_ (cm^–1^)	*T*_v_ (cm^–1^)	*R*_e_ (Å)	ω_e_ (cm^–1^)	*B*_e_ (cm^–1^)	*D*_e_ (cm^–1^)	μ_e_ (a.u)
X^1^Σ^+^	0^a^	0^a^	1.881^a^	860.7^a^	0.589^a^	28,619^a^	1.567^a^
	0^b^		1.880^b^	902.0^b^			
(1)^3^Π	4524^a^	6378^a^	2.056^a^	617.6^a^	0.493^a^	13,075^a^	0.043^a^
			2.050^b^	606.9^b^			
(1)^1^Π	5633^a^	7477^a^	2.051^a^	648.4^a^	0.496^a^	22,987^a^	0.064^a^
			2.090^b^	682.6^b^			
(1)^3^Σ^+^	20,006^a^	21,029^a^	2.022^a^	588.2^a^	0.509^a^	19,669^a^	0.011^a^
(2)^1^Σ^+^	27,816^a^	28,143^a^	1.956^a^	645.9^a^	0.544^a^	796^a^	0.335^a^
(2)^3^Σ^+^	28,697^a^	30,825^a^	2.073^a^	614.8^a^	0.485^a^	10,992^a^	0.570^a^
(1)^3^Δ	29,610^a^	32,598^a^	2.116^a^	558.8^a^	0.467^a^	20,654^a^	0.678^a^
(2)^3^Π	31,220^a^	47,429^a^	2.913^a^	350.7^a^	0.246^a^	8382^a^	0.080^a^
(2)^3^Σ^-^	32,274^a^	38,868^a^	2.210^a^	555.3^a^	0.426^a^	7416^a^	0.474^a^
(1)^5^Π	35,006	44,923^a^	2.460^a^	305.1^a^	0.345^a^	4228^a^	1.637^a^
(3)^3^Π	38,509^a^	48,873^a^	2.705^a^	193.5^a^	0.285^a^	1169^a^	1.024^a^
(4)^3^Π	47,108^a^	51,722^a^	2.482^a^	170.2^a^	0.335^a^	3514^a^	0.998^a^
(2)^3^Δ	50,271^a^		3.081^a^	138.4^a^	0.219^a^	419^a^	0.254^a^
(4)^1^Π	53,730^a^	55,085^a^	2.036^a^	592.7^a^	0.503^a^	4021^a^	2.281^a^

a Present work using MRCI + Q at the spin-free level.

b Reference (15).

In each molecule, the ground state X^1^Σ^+^ seems to bear the largest *D*_e_ value compared to higher excited states. Among the two molecules, one can notice that the dissociation energy *D*_e_ for the ground state decreases from 28,619 cm^–1^ in BeSe to 24,565 cm^–1^ in BeTe. It is clear that the potential energy curves exhibit a behavior of avoided crossing between (1)^1^Δ and (2)^1^Δ states of both molecules, between the (4)^1^Π and (5)^1^Π states of BeSe and (2)^1^Π and (3)^1^Π states of BeTe. Such a behavior can be explained by the non-crossing rule where non-adiabatic couplings occur between the adiabatic states.

The null value of dipole moments at large internuclear distances (for most electronic states) is evidence that these molecules dissociate into neutral atoms at the asymptotic limit. In particular, the ground states X^1^Σ^+^ of both molecules have a covalent character at the dissociation limit and a mixed character at smaller internuclear separations. The dipole moment curve (DMC) of this state presents a maximum magnitude |μ| = 1.58 a.u at *R* = 2.08 Å for BeSe and |μ| = 1.33 a.u at *R* = 2.24 Å for BeTe. A type of confirmation of the mixed covalent/ionic character of the ground state can be obtained by calculating the percentage ionic character using the formula (23) at the equilibrium position. For the ground state X^1^Σ^+^, the ionicity is found to be *f*_ionic_ = 0.44 for BeSe and 0.34 for BeTe. This verifies that the covalent character dominates over the ionic character around the equilibrium position of the ground state of BeSe and BeTe molecules.

On the other hand, the spin-free curves investigated using the basis set ECP28MDF and ECP46MDF for Se and Te, respectively, and by introducing *f* function in the basis set of Be are provided in Figures FS5 and FS6 in the Supporting Information and their corresponding states with the spin-orbit effect are given in figures (FS7, FS8). For the (1)0^+^ state, the dominant SΛ component is 99.05% and 94.16% X^1^Σ^+^ for BeSe and BeTe, respectively. For the (1)2 and (1)0^–^ states, the main parent SΛ is (1)^3^Π with percentage compositions of 99.99% and 100% for BeSe and 99.98% and 100% for BeTe. The dominant component for (2)0^–^ and (3)1 states is the (1)^3^Σ^–^ state. Concerning the (1)1, (2)0^+^, (2)1, and (3)0^+^ states, the main compositions are 80.49% (1)^3^Π, 96.24% (1)^3^Π, 80.5% (1)^1^Π, and 99.39% (2)^1^Σ^+^, respectively, for BeSe. For BeTe, the dominant component for (1)1, (2)0^+^, and (2)1 states is 67.83% (1)^3^Π, 89.44% (1)^3^Π, and 67.91% (1)^1^Π, respectively.

### 3.2 Spectroscopic Constants and the Ro-Vibrational Calculations

By fitting the energy data of the ground and excited states calculated at the spin-free level using the ECPMWB basis set for Se and Te around their equilibrium position *R*_e_ into a polynomial in terms of the internuclear distance, the spectroscopic constants *T*_e_, *R*_e_, ω_e_, and *B*_e_ have been calculated for the investigated bound states of BeSe and BeTe and are presented in Table 2. The dissociation energies *D*_e,_ the dipole moments at the equilibrium position of these electronic states, and the vertical transition energy *T*_v_ are also calculated. The absence of spectroscopic constants of some electronic states is referred to the presence of avoided crossing near the minima of these states. Because the harmonic frequency ω_e_ is directly proportional to the vibrational energy *E*_v,_ then for deep states, probability of reaching the higher number of vibrational levels ω_e_ is larger, unlike the shallow states that possess smaller values of ω_e_.

As a confirmation of the reliability of our method given in Table 2, the trend of the spectroscopic constants is reported in Table 3, where *T*_e_, ω_e,_ and *B*_e_ decrease for each electronic state with the decrease in the electronegativity and *R*_e_ increases as we go from BeSe to BeTe.

**Table 3:**
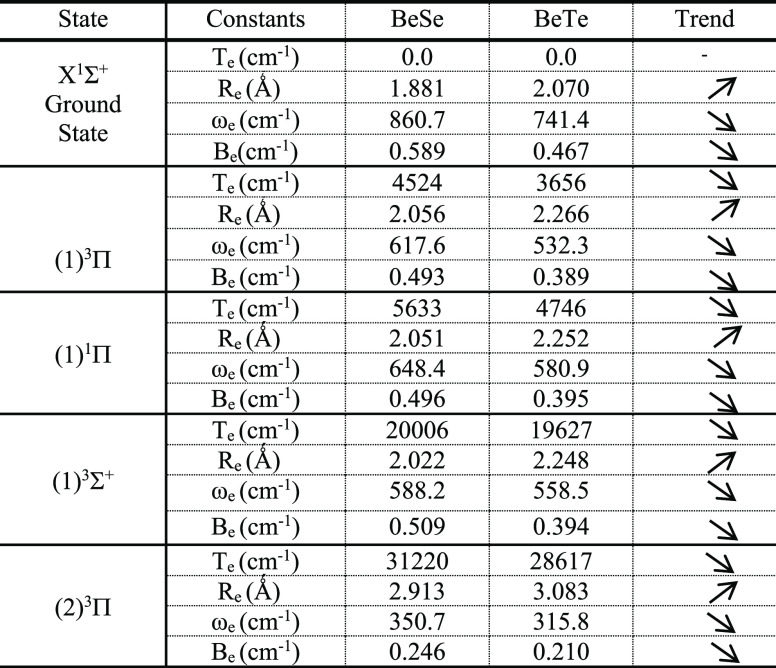
Study of the Trend of the Spectroscopic Constants of the Different Electronic States of the Molecules BeSe and BeTe

Additionally, the spectroscopic constants of BeSe calculated in the present work approach those given by Larbi et al.^15^ where the relative discrepancy for *R*_e_ of the ground state is 0.0% and that of ω_e_ is 4.5%. Also, these values are compared for the first two excited states in Table 2. However, the spectroscopic constants of the states investigated at the spin-orbit level using the ECP28MDF and ECP46MDF for Se and Te, respectively, are provided in Table TS1 in the Supporting Information.

The vibrational energy *E*_v_, the rotational constant *B*_v_, the centrifugal distortion constant *D*_v_, and the abscissas of the turning point *R*_min_ and *R*_max_ were calculated and are reported for the states investigated using the ECPMWB for Se and Te at the spin-free level in Tables TS2 and TS3 in the Supporting Information for several vibrational levels of many electronic states of BeSe and BeTe by using the canonical function approach^24^ and cubic spline interpolation between every two consecutive points of the potential energy curve. The vibrational, rotational study is absent for the other electronic states due to the failure of the canonical function approach in the cases of crossings and avoided crossings.

### 3.3 Radiative Lifetime and the Transition Dipole Moment Curves

**Figure 5:**
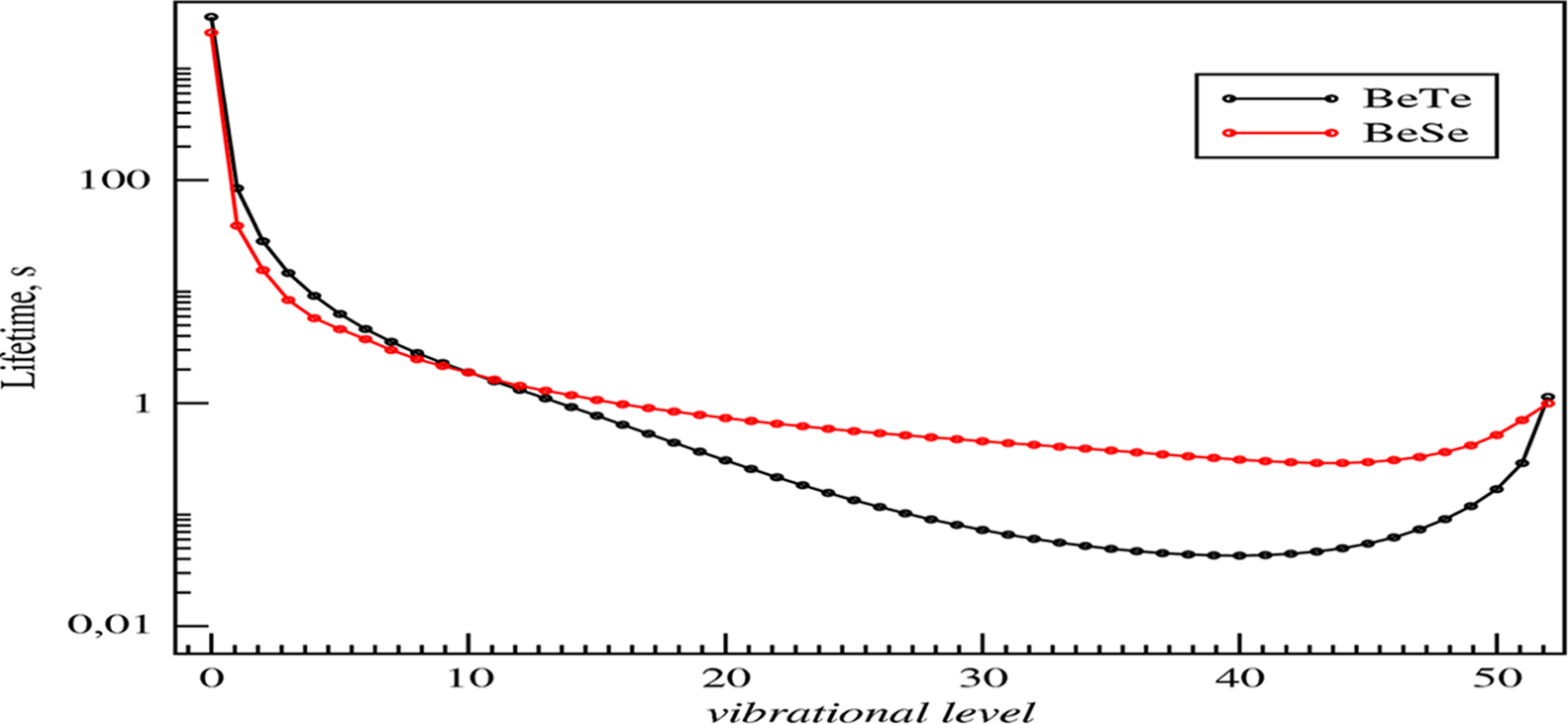
Radiative lifetimes of the vibrational levels of the ground state for BeTe and BeSe molecules investigated using ECP28MWB and ECP46MWB for Se and Te, respectively.

The measurement of the radiative lifetimes is of great interest for physicists working in fields like atomic physics, plasma physics, and astrophysics.^25−27^ This section is dedicated to investigating the radiative transition probabilities (Einstein A coefficients) and radiative lifetimes of all the ground-state vibrational levels for BeTe and BeSe molecules. These lifetimes are calculated using the ground-state potential energy curves and the permanent dipole moment curves determined previously and are presented in Figure 5.

The radiative lifetime of a vibrational state is given using the following expression
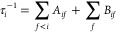
where *A*_*if*_ is the Einstein coefficient describing the probability of spontaneous emission from the vibrational state *i* to the lower energy state *f*.
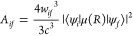
*w*_*if*_ = |*E*_*f*_ – *E*_*i*_| is the transition frequency between two states ψ_*i*_ and ψ_*f*_ and ⟨ψ_*i*_|μ(*R*)|ψ_*f*_⟩is the vibrational transition dipole moment between the initial state (*i*) and the final state (*f*). The black-body radiation coming from the surrounding environment at *T* = 300 K can induce stimulated absorption and emission processes, which are described using the Einstein coefficient *B*_*if*_ = *A*_*if*_*N*(ω_*if*_), where the number of black-body photons is
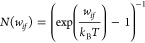


**Figure 6:**
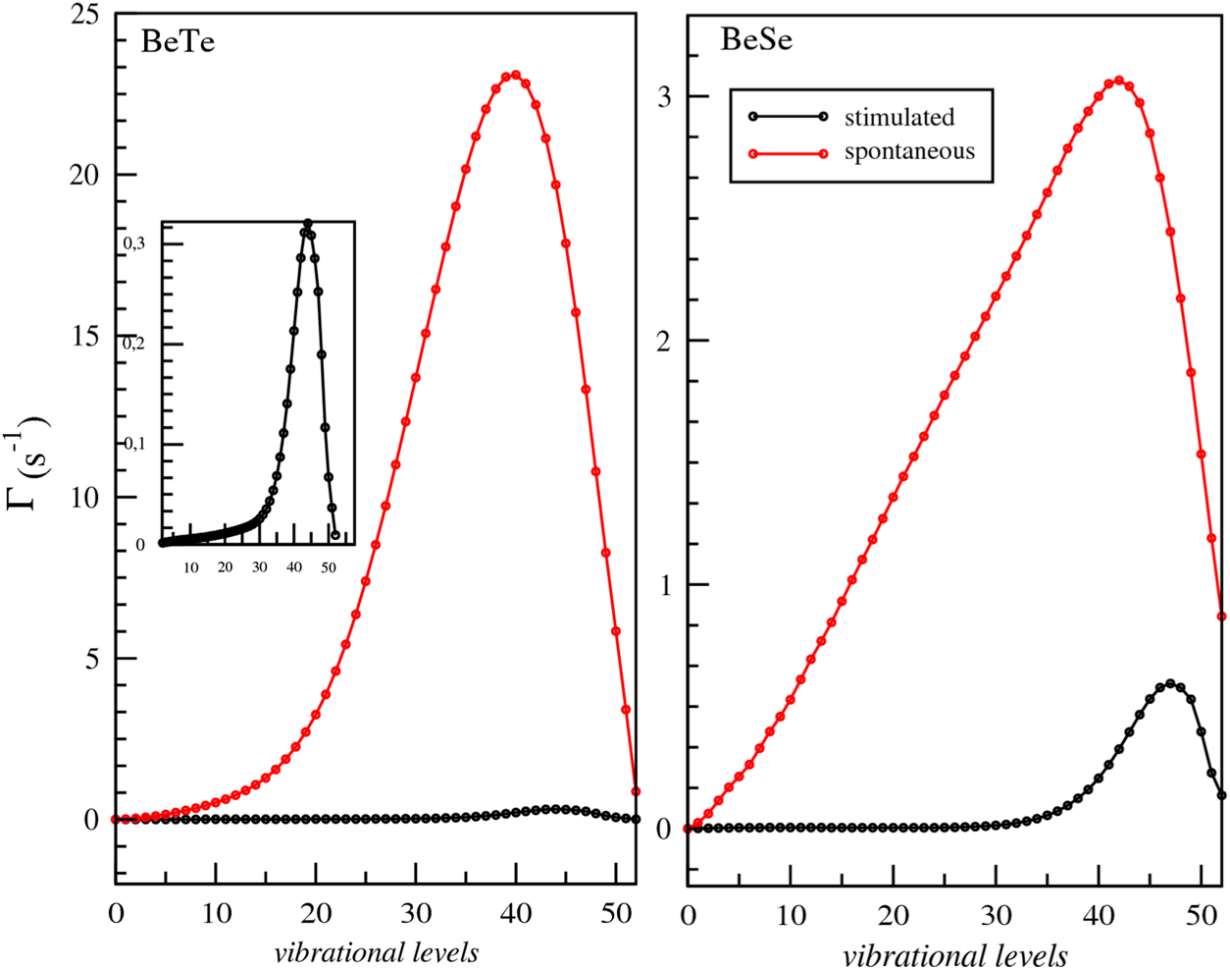
Spontaneous (red) and black-body radiation-stimulated (black) transition rates for the vibrational levels of the ground electronic state of BeTe and BeSe molecules.

Einstein coefficients sufficiently large to be relevant for analyzing their possible transition pathways are shown in Figure 6.

We found that the ground vibrational state *v* = 0 has lifetimes τ = 2905.95 and τ = 2098.83 s for BeTe and BeSe, respectively, as reported in Table 4.

**Table 4:** Radiative Lifetimes of the Vibrational Levels of the Ground State Investigated Using ECP28MWB and ECP46MWB for BeSe and BeTe Molecules

*V*	BeTe	BeSe
0	2905.95	2098.83
1	84.64	38.94
2	28.26	15.60
3	14.71	8.41
4	9.17	5.785
5	6.30	4.60
6	4.62	3.75
7	3.54	3.00
8	2.81	2.48
9	2.29	2.16
10	1.89	1.88
11	1.57	1.62
12	1.32	1.43
13	1.10	1.29
14	0.92	1.17
15	0.77	1.07
16	0.64	0.98
17	0.53	0.90
18	0.44	0.84
19	0.37	0.78
20	0.31	0.73
21	0.26	0.69
22	0.22	0.65
23	0.18	0.62
24	0.16	0.59
25	0.14	0.56
26	0.12	0.54
27	0.10	0.51
28	0.09	0.49
29	0.08	0.47
30	0.07	0.46
31	0.06	0.44
32	0.06	0.42
33	0.05	0.41
34	0.05	0.40
35	0.05	0.38
36	0.04	0.36
37	0.04	0.35
38	0.04	0.33
39	0.04	0.32
40	0.04	0.31
41	0.04	0.30
42	0.04	0.29
43	0.05	0.29
44	0.05	0.29
45	0.06	0.30
46	0.06	0.31
47	0.07	0.33
48	0.09	0.36
49	0.12	0.42
50	0.17	0.52
51	0.29	0.70
52	1.14	0.99

In terms of trends, the lifetimes for BeSe and BeTe are initially significant at the lowest vibrational levels, decrease with increasing vibrational levels, and start to increase again starting from vibrational levels where peaks in spontaneous and stimulated rates can be distinguished. The shortest lifetime for both molecules BeTe and BeSe corresponds to the peak of the stimulated transition rate, which is also close to the maximum of the spontaneous transition rate. This behavior is similar to that of previously investigated diatomic molecules, consisting of alkali–alkaline earth atoms.^28^ For BeTe, the peak of the stimulated transition can be distinguished in the inset presented in the same figure. For higher vibrational levels, both spontaneous and stimulated rates monotonically decrease because the transition frequencies ω_*if*_ between the highly excited vibrational states become lower as the highly excited states are energetically closer than those of the lower-energy states. The calculation of the vibrational lifetimes of the excited states takes into account two possible transitions: bound–bound and bound–free transitions. The radiative lifetime of a vibrational level υ′ corresponding to only bound–bound transitions is given using the following expression
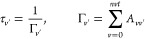
*A*_*vv*′_ is the Einstein coefficient taking, for example, the transition between 2 ^1^∑^+^(*v*′) and X ^1^∑^+^ (*v*) level states.

It has been shown previously, by Zemke et al.,^29^ that there is a missing contribution in the radiative lifetimes. It corresponds to the bound–free transitions, which is more significant for the higher vibrational levels close to the dissociation limit of the excited electronic states, which are found in the form of the continuum radiation to states above the dissociation limit of the ground state. It matches the contribution of the *bound–free* transition missed in the equation above. It is related to the transition between the vibrational level *v*′, which belongs to the excited electronic state to the continuum of the ground state or the lower state in general. Such contribution is not negligible as it was demonstrated by Zemke et al.,^29^ Partridge et al.,^30^ and Berriche et al.^31^ This term is more obvious for the higher vibrational levels due the difference in location of the potential wells. This contribution is calculated using two different approximations: “Franck–Condon”^30,31^ and “*sum rule*”^32,33^ approach.

#### 3.3.1 Franck–Condon Approximation

This approximation, which was proposed by Zemke et al.,^29^ gives the bound–free contribution as
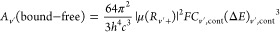
where Δ*E*_υ′,cont_ = *E*_υ′_ – *E*_as_ is the energy difference between the vibrational level υ′ and the energy of the asymptotic limit of the lower electronic state, to which belongs the continuum. The quantity μ(*R*_υ′+_) corresponds to the transition dipole moment at the right external turning point of the vibrational level *v*′.
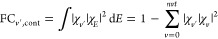


#### 3.3.2 Sum Rule Approximation

Pazyuk et al.^32,33^ have implemented this approach. It allows reproducing the radiative lifetime components for diatomic vibronic states. In addition, this approximation has a high efficiency for non-diagonal systems and particularly for those with significant continuum contributions. The radiative lifetime using the approximate sum rule is given using the following expression
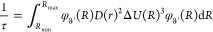
φ_υ′_(*R*) is the wave function of the vibrational level belonging to the (2)^1^∑^+^ excited electronic state. *D*(*R*) is the transition dipole moment between the ground X^1^∑^+^ and first excited state (2)^1^∑^+^. Δ*U*(*R*) is the energy difference between the ground X^1^∑^+^ state and the first excited state (2)^1^∑^+^. The Franck–Condon approximation and sum rule approximation of radiative lifetimes corresponding to the X^1^Σ^+^–(2)^1^Σ^+^ transition of BeSe are given in Figure FS9 in the Supporting Information, while the corresponding values of lifetimes for this transition are reported in Table TS4.

**Figure 7:**
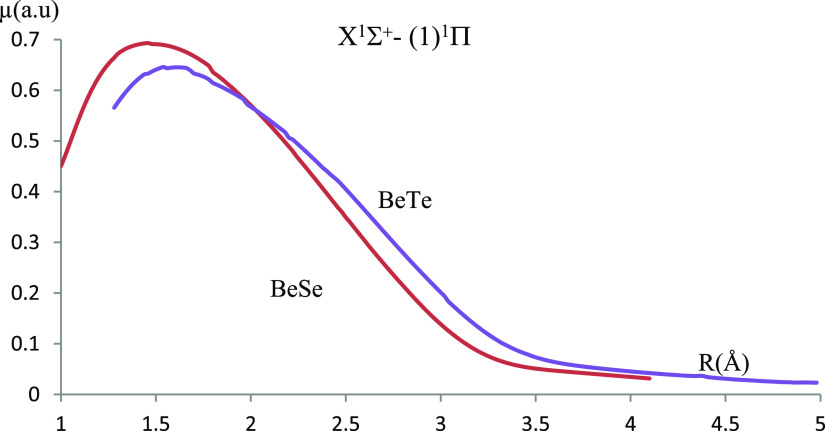
Transition dipole moment curves of the X^1^Σ^+^–(1)^1^Π transition investigated using ECP28MWB and ECP46MWB for Se and Te, respectively.

Additionally, the transition dipole moment curves are investigated and given in Figure 7 for the X^1^Σ^+^–(1)^1^Π transition of BeSe and BeTe and in Figure FS10 in the Supporting Information for the X^1^Σ^+^–(2)^1^Σ^+^ transition of BeSe molecules.

## 4 Conclusions

The potential energy curves and dipole moment curves of the ground and excited electronic states were investigated for the molecules BeSe and BeTe, respectively. The calculations were performed by employing the MCSCF/MRCI technique, similar to our previously published work.^34−36^ The current study was performed at the spin-free and spin-orbit coupling level. The spectroscopic constants *T*_e_, *R*_e_, ω_e_, and *B*_e_ and the dissociation energy *D*_e_ have been calculated for most of the bound states. The spectroscopic parameters of the ground and excited states of BeSe and BeTe show a very good agreement. A ro-vibrational study was performed using the canonical function approach and cubic spline interpolation to find the ro-vibrational constants *E*_v_, *B*_v_, and *D*_v_ with the abscissas of turning points *R*_min_ and *R*_max_ for many electronic states. Finally, the radiative transition probabilities (Einstein A coefficients) and radiative lifetimes of all the ground-state vibrational levels for BeTe and BeSe molecules were calculated. Up to our knowledge, this is the first work displaying the molecule BeTe in the literature.

## Supporting Information Available

Potential energy curves of the quintet states of BeSe and BeTe molecules; permanent dipole moment curves of the quintet states of BeSe and BeTe molecules; potential energy curves of BeSe and BeTe molecules; potential energy curves of the Ω = 0^±^, 1, and 2 states of BeSe and BeTe molecules; Franck–Condon approximation and sum rule approximation; transition dipole moment curves of the X^1^Σ^+^–(2)^1^Σ^+^ transition of BeSe molecules; spectroscopic parameters of the Ω = 0^±^, 1, and 2 states of BeSe and BeTe molecules; values of the eigenvalue *E*_v_, the rotational constant *B*_v_, the centrifugal distortion constant *D*_v_, and *R*_min_ and *R*_max_ of the different electronic states of the BeSe and BeTe molecules; and radiative lifetimes of the vibrational levels of the X^1^Σ^+^–(2)^1^Σ^+^ transition of BeSe (PDF)
