# Supplementary material for: Electronic Structures and Transition Properties of BeSe and BeTe Molecules
Source: ACS Omega. 2021 Nov 7;6(45):30335–43. doi: 10.1021/acsomega.1c03170 (PMC8603188; doi:10.1021/acsomega.1c03170)
Supplement: Supplementary file 1 — ao1c03170_si_001.pdf [file ao1c03170_si_001.pdf]

Supplementary Information for Manuscript :
Electronic structure and transition properties of BeSe and BeTe molecules

Israa Zeid[1], Nayla El-Kork* [2] Mohamed Farjallah [3], Hela Ladjimi [3], Hamid Berriche [3,4]

and Mahmoud Korek[1]

[1]Faculty of Science, Beirut Arab University, P.O. Box 11-5020, Beirut 1107 2809, Lebanon.

[2]Khalifa University, Physics department P.O. Box 127788, Abu Dhabi, UAE.

[3]Laboratory of Interfaces and Advanced Materials, Faculty of Science, University of Monastir, 5019 Monastir, Tunisia.

[4]Department of Mathematics and Natural Sciences, School of Arts and Sciences,
American University of Ras Al Khaimah, RAK, P.O. Box 10021, UAE.

Keywords: ab initio calculation; potential energy curves; spectroscopic constants; rovibrational calculation; dipole moment.

Submitted to: ACS Omega

----

*Author to whom correspondence should be addressed
email: nayla.elkork@ku.ac.ae

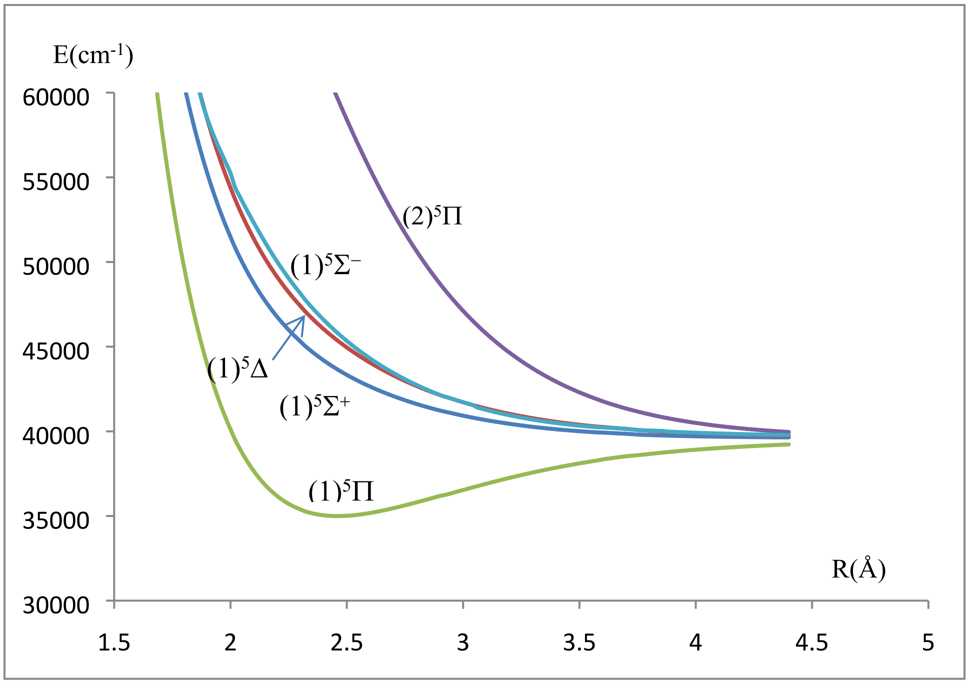

Figure FS1: Potential energy curves of the quintet states of BeSe molecule investigated using the ECP28MWB for Se.

.

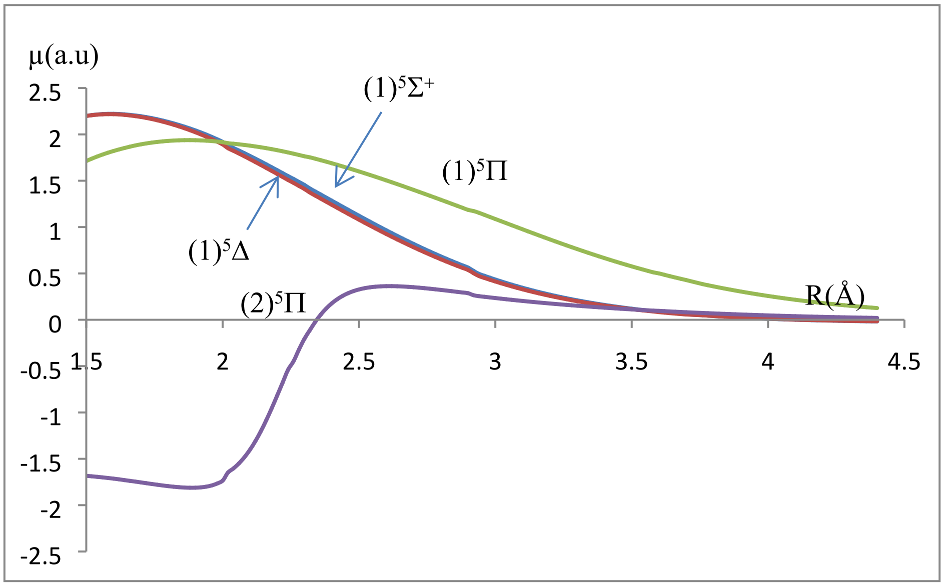

Figure FS2: Permanent dipole moment curves of the quintet states of BeSe molecule investigated using the ECP28MWB for Se.

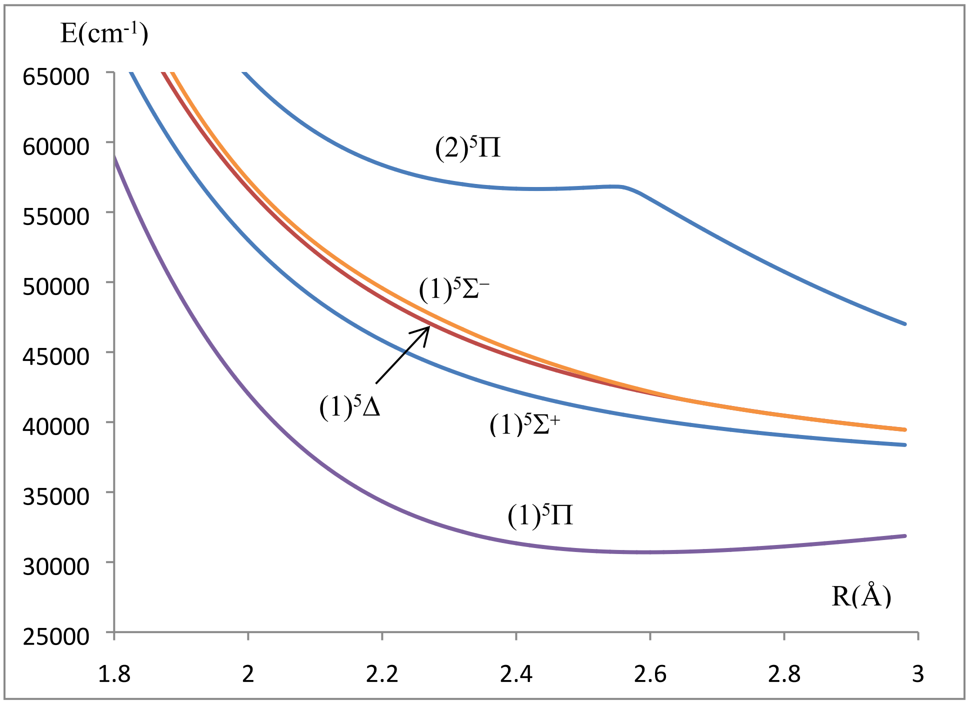

Figure FS3: Potential energy curves of the quintet states of BeTe molecule investigated using the ECP46MWB for Te.

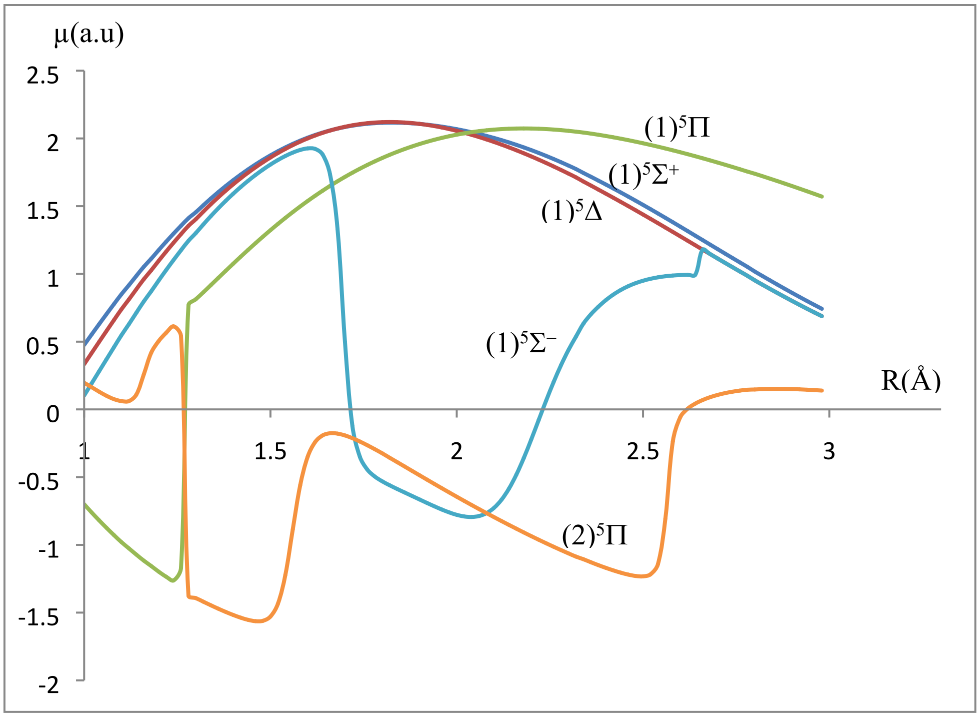

Figure FS4: Permanent dipole moment curves of the quintet states of BeTe molecule investigated using the ECP46MWB for Te.

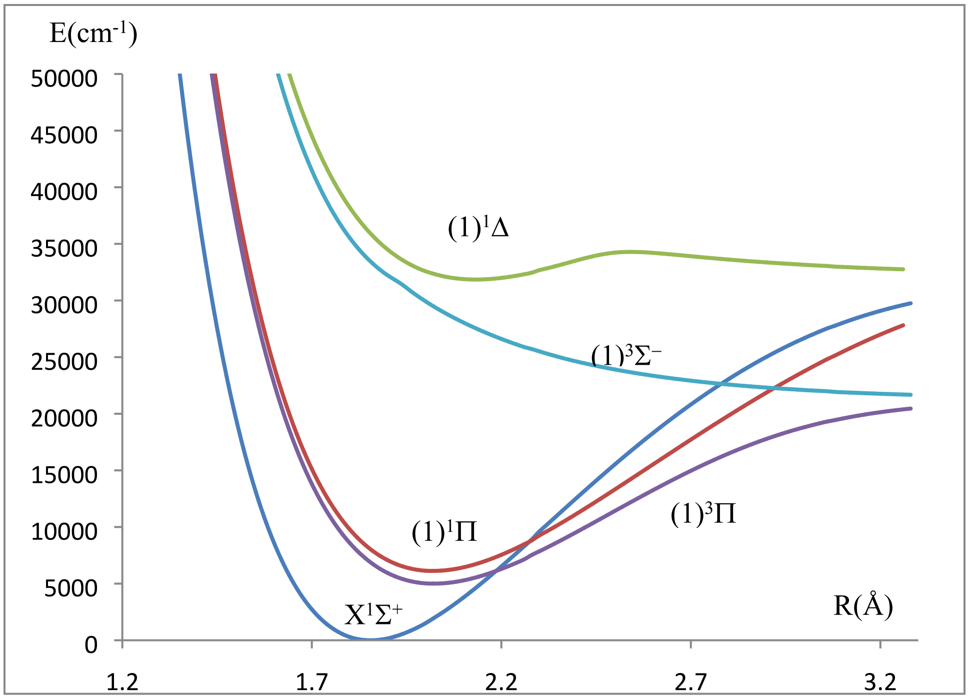

Figure FS5: Potential energy curves of BeSe molecule using the ECP28MDF for Se.

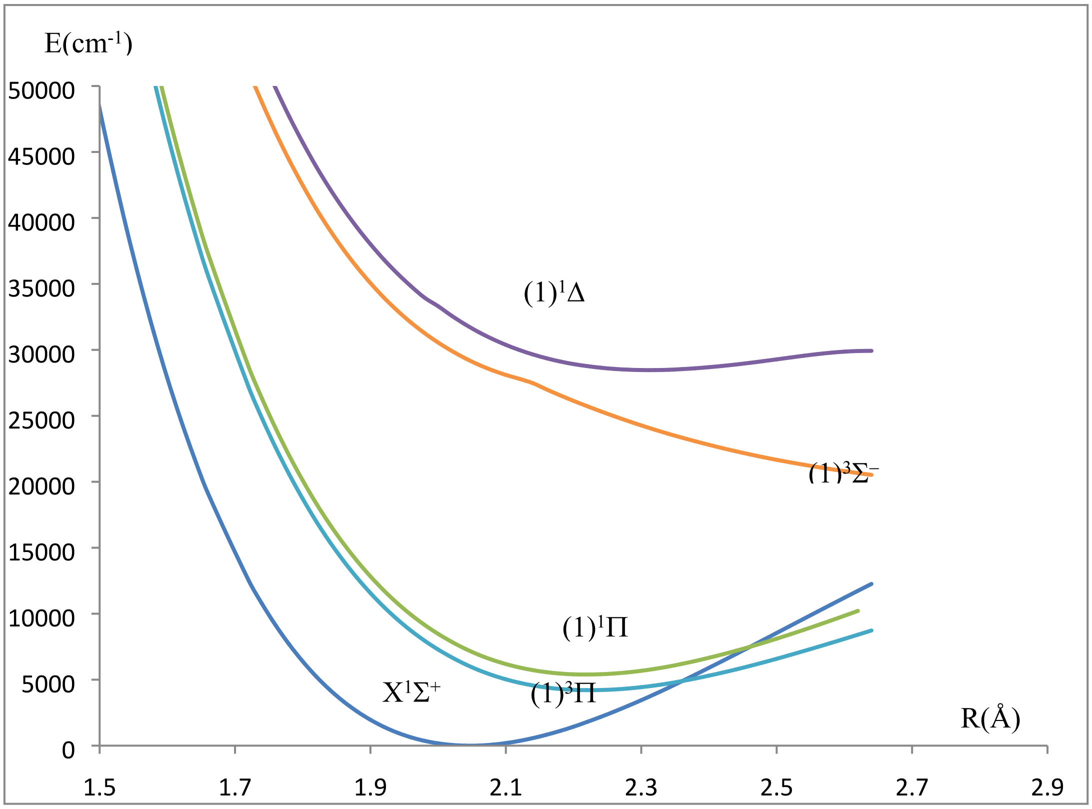

Figure FS6: Potential energy curves of BeTe molecule using ECP46MDF for Te.

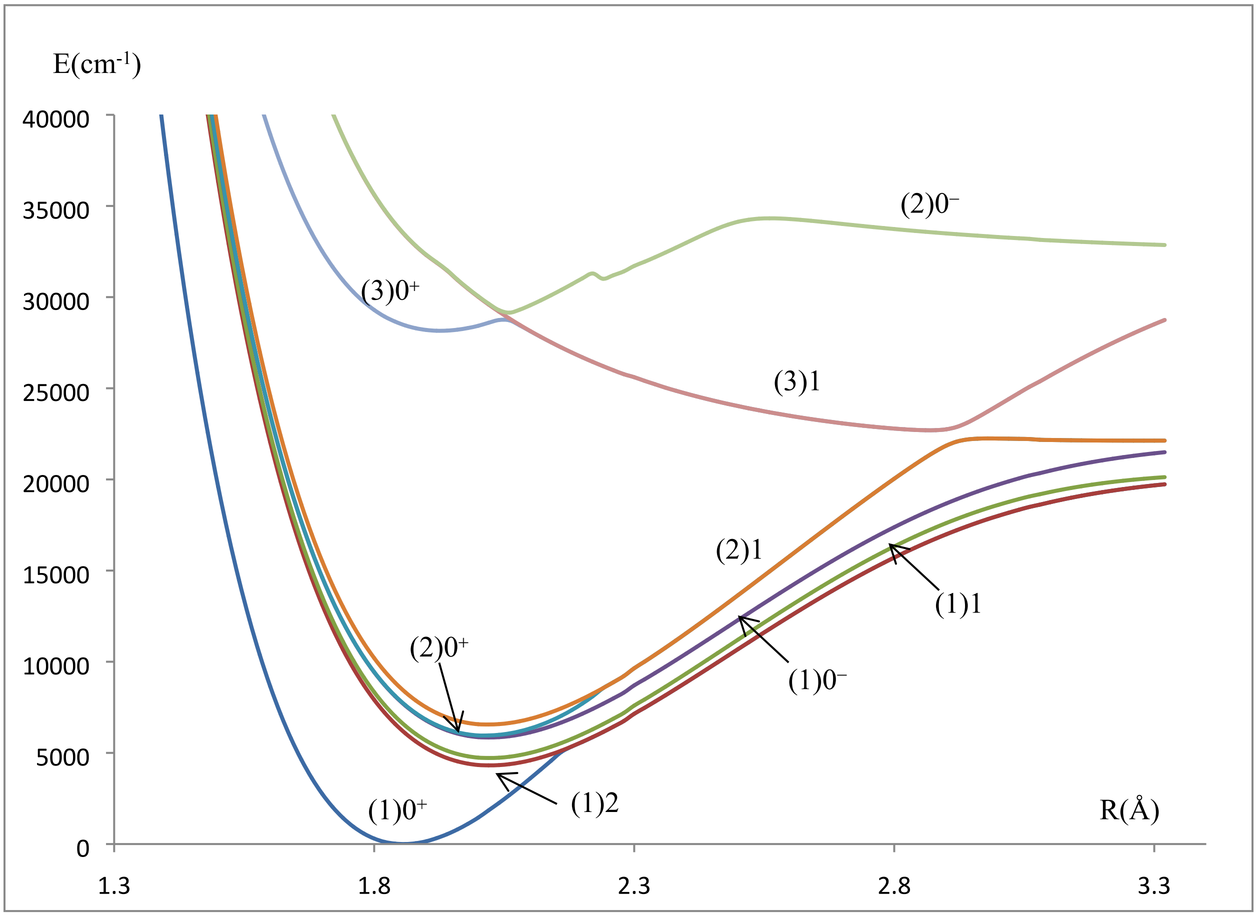

Figure FS7: Potential energy curves of the Ω= 0 ± ,1,2 states of BeSe molecule.

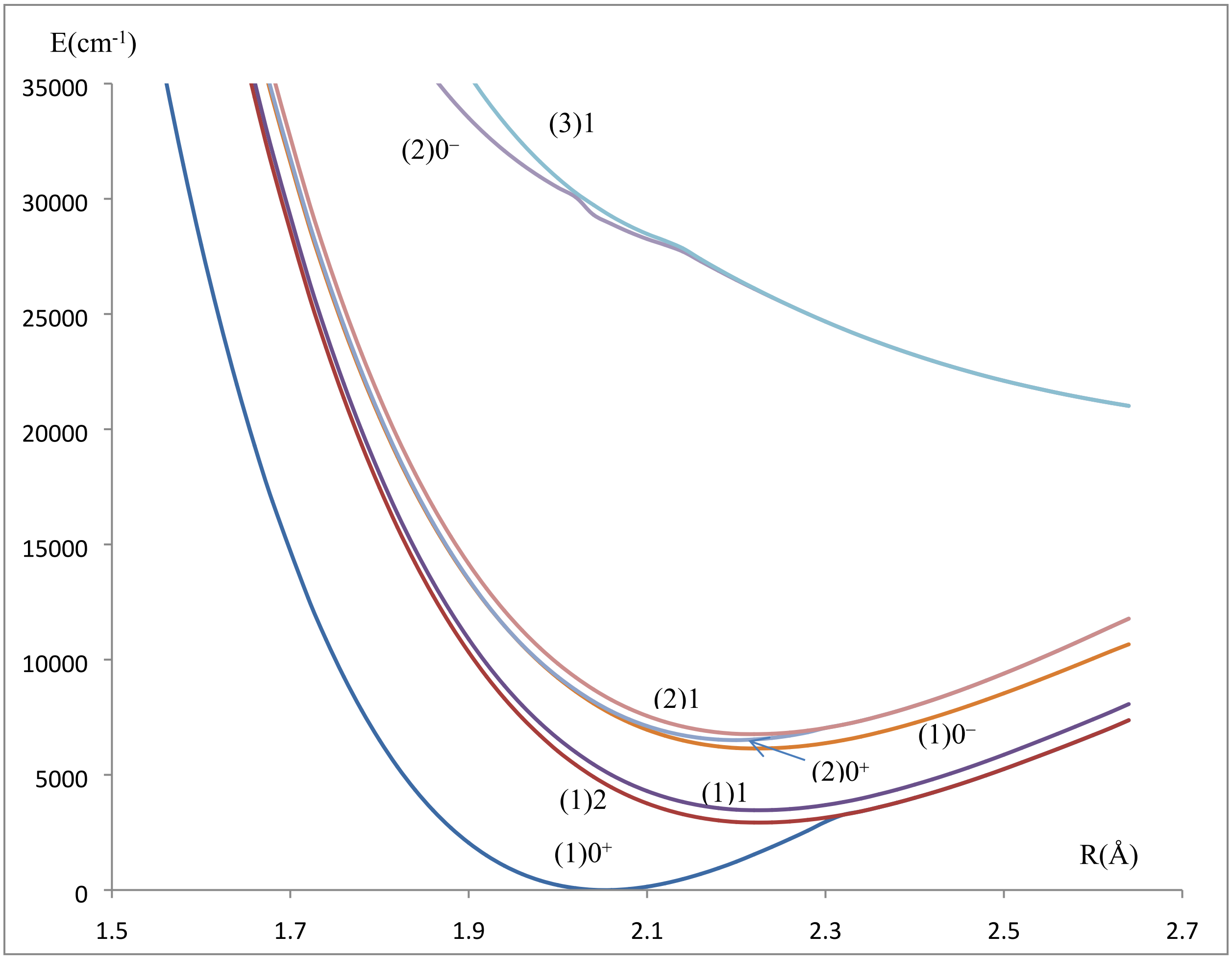

Figure FS8: Potential energy curves of the Ω= $0^{\pm}$,1,2 states of BeTe molecule.

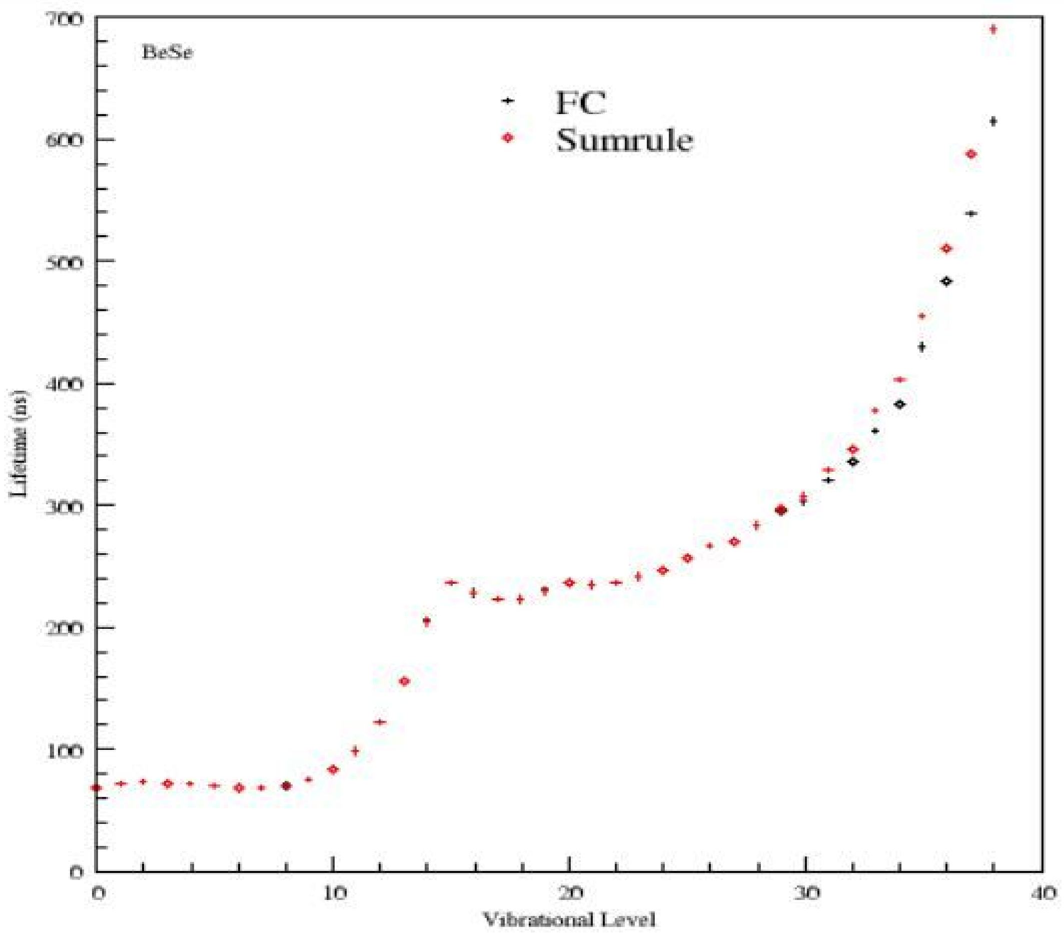

Figure FS9: The Franck-Condon approximation and sum rule approximation of radiative lifetimes corresponding to $X^1\Sigma$+- $(2)^1\Sigma^+$ transition of BeSe investigated using ECP28MWB Se.

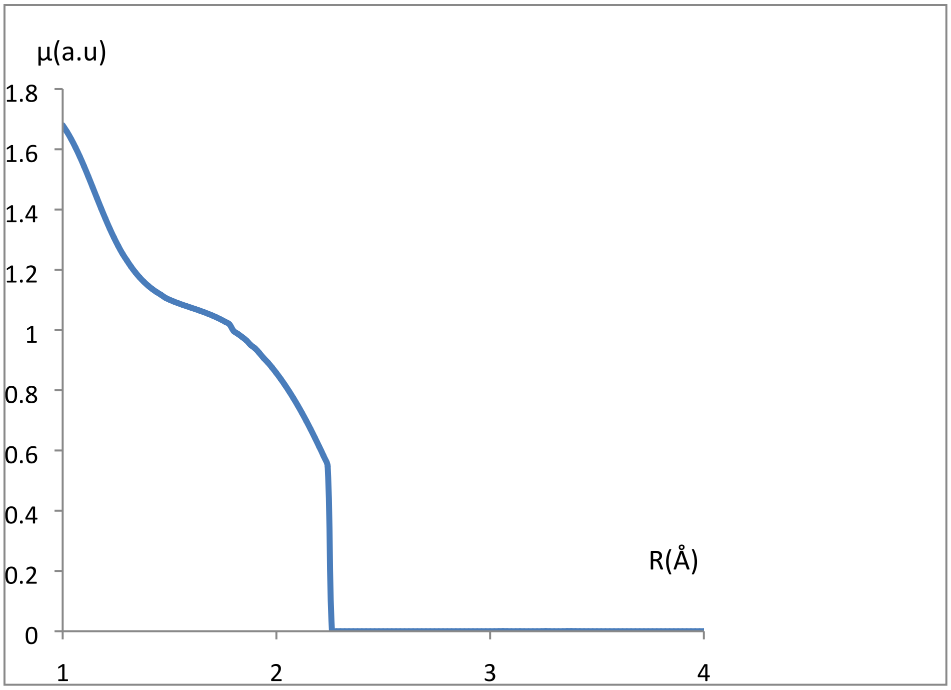

Figure FS10: The transition dipole moment curve of the $X^1\Sigma^+$- $(2)^1\Sigma^+$ transition of BeSe molecule investigated using ECP28MWB for Se.

Table TS1: The spectroscopic parameters of the $\Omega = 0^{\pm}, 1, 2$ states of BeSe and BeTe molecules.

	State	T_e (cm^{-1})	R_e (Å)	ω_e (cm^{-1})	B_e (cm^{-1})
BeSe	$(1)0^+$	0	1.856	863.1	0.6050
	(1)2	4315	2.022	676.5	0.5103
	(1)1	4719	2.022	674.2	0.5101
	$(1)0^-$	5845	2.021	673.0	0.5106
	$(2)0^+$	5954	2.014	697.2	0.5141
	(2)1	6557	2.019	688.5	0.5114
	$(3)0^+$	22677	2.859	632.8	0.2556
	State	T_e (cm^{-1})	R_e (Å)	ω_e (cm^{-1})	B_e (cm^{-1})
BeTe	$(1)0^+$	0	2.053	753.9	0.4749
	(1)2	2927	2.227	586.0	0.4038
	(1)1	3463	2.226	592.4	0.4041
	$(1)0^-$	6143	2.225	593.1	0.4046
	$(2)0^+$	6509	2.199	682.8	0.4139
	(2)1	6765	2.220	614.5	0.4063

Table TS2: Values of the eigenvalue E_v, the rotational constant B_v, the centrifugal distortion constant D_v and R_{min} and R_{max} of the different electronic states of the molecule BeSe investigated using ECP28MWB for Se.

$X^1\Sigma^+$						$(1)^1\Pi$					
v	E (cm^{-1})	B_v(cm^{-1})	D_v x10^6 (cm^{-1})	R_{min}(Å)	R_{max}(Å)	v	E_v(cm^{-1})	B_v(cm^{-1})	D_v x10^6 (cm^{-1})	R_{min} (Å)	R_{max} (Å)
0	433.05	0.588	1.1726	1.809	1.954	0	323.52	0.494	1.1571	1.974	2.135
1	1260.08	0.582	1.1109	1.772	2.014	1	965.34	0.490	1.1575	1.924	2.203
2	2087.58	0.577	1.2382	1.740	2.059	2	1599.94	0.486	1.1587	1.891	2.254
3	2896.03	0.574	1.3447	1.717	2.097	3	2227.31	0.482	1.1421	1.866	2.297
4	3683.90	0.570	1.1553	1.698	2.132	4	2849.03	0.478	1.1377	1.845	2.335
5	4467.03	0.565	1.1813	1.681	2.163	5	3464.92	0.474	1.1566	1.827	2.371
6	5242.06	0.560	1.2366	1.667	2.193	6	4073.53	0.470	1.1261	1.810	2.405
7	6007.09	0.556	1.3156	1.654	2.223	7	4676.32	0.466	1.1500	1.796	2.438
8	6759.93	0.552	1.2556	1.642	2.251	8	5272.20	0.462	1.1322	1.783	2.469
9	7502.68	0.546	1.0809	1.631	2.278	9	5861.73	0.458	1.1295	1.771	2.499
10	8240.33	0.541	1.3152	1.621	2.305	10	6444.77	0.454	1.1226	1.760	2.530
11	8967.29	0.538	1.4180	1.611	2.332	11	7021.38	0.450	1.1504	1.750	2.559
12	9681.54	0.533	1.0794	1.603	2.358	12	7591.10	0.447	1.1587	1.740	2.588
13	10389.56	0.527	1.1819	1.595	2.383	13	8154.10	0.443	1.1802	1.731	2.616
14	11089.51	0.523	1.5050	1.587	2.409	14	8710.06	0.439	1.1319	1.722	2.644
15	11776.33	0.519	1.2469	1.579	2.434	15	9259.72	0.435	1.1373	1.714	2.672

(1)$^3\Pi$					
v	E_v(cm^{-1})	B_v(cm^{-1})	D_v x10^6 (cm^{-1})	R_{min}(Å)	R_{max}(Å)
0	308.51	0.491	1.2471	1.979	2.143
1	920.94	0.486	1.2382	1.927	2.213
2	1526.65	0.482	1.2090	1.895	2.265
3	2127.08	0.477	1.2378	1.870	2.311
4	2718.78	0.472	1.2921	1.849	2.352
5	3299.40	0.467	1.2444	1.831	2.391
6	3872.01	0.463	1.2636	1.816	2.428
7	4435.40	0.457	1.2943	1.801	2.464
8	4989.01	0.453	1.3470	1.789	2.499
9	5532.09	0.447	1.3288	1.777	2.533
10	6065.63	0.443	1.3478	1.766	2.567
11	6589.40	0.437	1.4165	1.756	2.601
12	7102.02	0.432	1.4435	1.747	2.635
13	7603.27	0.426	1.3882	1.738	2.669
16	9802.78	0.431	1.1268	1.707	2.699
17	10339.27	0.427	1.1416	1.699	2.727

(1)$^3\Sigma^+$					
v	E_v(cm^{-1})	B_v(cm^{-1})	D_v x10^6 (cm^{-1})	R_{min} (Å)	R_{max} (Å)
0	294.49	0.532	5.7115	1.942	2.111
1	879.91	0.504	1.3060	1.890	2.183
2	1460.22	0.499	1.4209	1.856	2.235

Table TS3: Values of the eigenvalue E_v, the rotational constant B_v, the centrifugal distortion constant D_v and R_{min} and R_{max} of the different electronic states of the molecule BeTe investigated using ECP46MWB for Te.

$(X)^1\Sigma^+$					
v	E_v(cm^{-1})	B_v(cm^{-1})	D_v x10^6 (cm^{-1})	R_{min} (Å)	R_{max}(Å)
0	369.63	0.4660	0.7456	1.999	2.147
1	1103.12	0.5901	0.5098	1.953	2.209
2	1828.11	0.5862	1.8304	1.922	2.255
3	2544.67	0.5758	3.9172	1.899	2.294
4	3253.13	0.5926	2.6823	1.879	2.329
5	3953.65	0.5750	3.3535	1.862	2.362
6	4646.27	0.5470	0.9055	1.847	2.394

$(1)^1\Pi$					
v	E_v(cm^{-1})	B_v(cm^{-1})	D_vx10^7 (cm^{-1})	R_{min}(Å)	R_{max}(Å)
0	290.56	0.3938	7.2587	2.172	2.338
1	868.28	0.3911	7.2253	2.119	2.408
2	1441.16	0.5192	2.7696	2.085	2.459
3	2008.90	0.5303	1.8395	2.058	2.503
4	2571.20	0.5419	1.0374	2.036	2.542
5	3127.92	0.5409	3.1821	2.017	2.578
6	3678.99	0.5662	1.8699	2.001	2.614
7	4224.34	0.5658	4.4948	1.986	2.647

TS4: Radiative lifetimes of the vibrational levels of the $X^1\Sigma^+$- $(2)^1\Sigma^+$ transition of BeSe investigated using ECP28MWB Se.

Vibrational Level	τ(FC)	τ(Sumrule)
0	68.77	68.77
1	71.35	71.34
2	72.81	72.81
3	72.13	72.12
4	70.75	70.73
5	69.20	69.19
6	68.12	68.11
7	67.90	67.89
8	69.70	69.68
9	74.63	74.59
10	83.74	83.65
11	98.57	98.42
12	121.49	121.26
13	156.12	155.86
14	205.33	205.07
15	236.00	235.73
16	227.74	227.51
17	222.19	221.98
18	223.23	223.04
19	230.32	230.14
20	236.64	236.48
21	234.95	234.80
22	235.78	235.65
23	241.41	241.29
24	246.29	246.18
25	257.02	256.92
26	266.02	265.97
27	269.87	269.84
28	282.74	283.26
29	295.79	297.10
30	304.01	307.06
31	319.91	328.38
32	335.85	345.98
33	359.84	376.57
34	382.47	402.17
35	429.53	453.93
36	483.89	510.88
37	538.49	587.77
38	614.53	690.33
